# Noninvasive positive-pressure ventilation in pregnancy to treat acute pulmonary edema induced by tocolytic agents: a case report

**DOI:** 10.1186/s13256-021-02704-w

**Published:** 2021-03-21

**Authors:** Kotaro Takahashi, Koji Nishijima, Masayuki Yamaguchi, Kensuke Matsumoto, Shunya Sugai, Takayuki Enomoto

**Affiliations:** Department of Obstetrics and Gynecology, Niigata University Medical and Dental Hospital, 1-757 Asahimachi-dori, Chuo-ku, Niigata, 951-8510 Japan

**Keywords:** Noninvasive ventilation, Pulmonary edema, Tocolytic agents, Preterm labor, Pregnancy

## Abstract

**Background:**

We report a case of pulmonary edema induced by tocolytic agents that was successfully managed with noninvasive positive-pressure ventilation (NPPV) and resulted in extended gestation.

**Case presentation:**

A 36-year-old Japanese pregnant woman received tocolytic therapy with ritodrine hydrochloride, magnesium sulfate, nifedipine, and betamethasone from 28 weeks of gestation. She developed respiratory failure. and her chest X-ray showed enlarged pulmonary vascular shadows. At 29 weeks and 1 day of gestation, she was diagnosed with pulmonary edema induced by tocolytic agents. Because respiratory failure worsened 2 days after ritodrine hydrochloride and magnesium sulfate were stopped, NPPV was initiated. Her respiratory status improved and she was weaned off of NPPV after 3 days. She underwent cesarean section because of breech presentation at 30 weeks and 0 days of gestation due to initiation of labor pains.

**Conclusions:**

NPPV can be safely administered in cases of tocolytic agent-induced pulmonary edema during pregnancy.

## Background

Acute pulmonary edema in pregnant women is an uncommon but life-threatening event that requires emergency care [1]. One cause of acute pulmonary edema is administration of tocolytic agents such as β-adrenergic receptor agonists and magnesium sulfate [2]. Therefore, in treatment of threatened preterm labor, development of acute pulmonary edema should be monitored.

Noninvasive positive-pressure ventilation (NPPV) is a form of noninvasive ventilation (NIV) that can be utilized for treatment of pulmonary edema, even in pregnancy [1]. However, few studies have investigated the efficacy of NPPV during pregnancy, and reports describing NPPV management for pulmonary edema due to tocolytic agents are scarce.

We report a case of hypoxemic respiratory failure due to pulmonary edema induced by tocolytic agents that was successfully and safely managed with NPPV and resulted in extended gestation.

## Case presentation

**Fig. 1:**
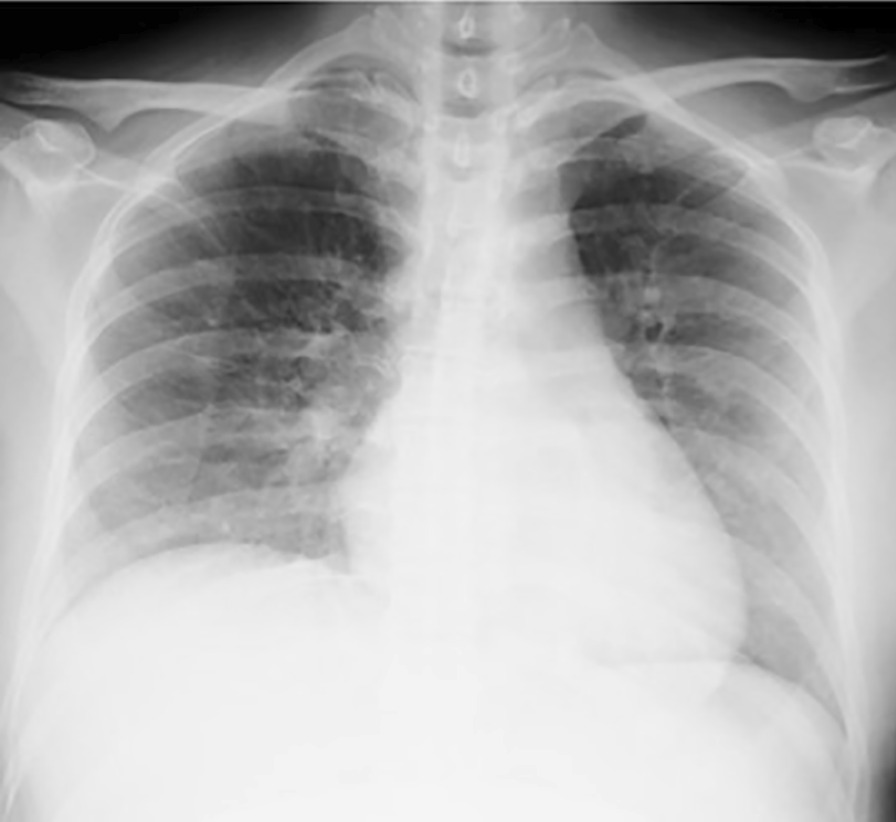
The chest X-ray taken in a sitting position after the patient developed dyspnea. Enlarged pulmonary vascular shadows indicated pulmonary edema

**Fig. 2:**
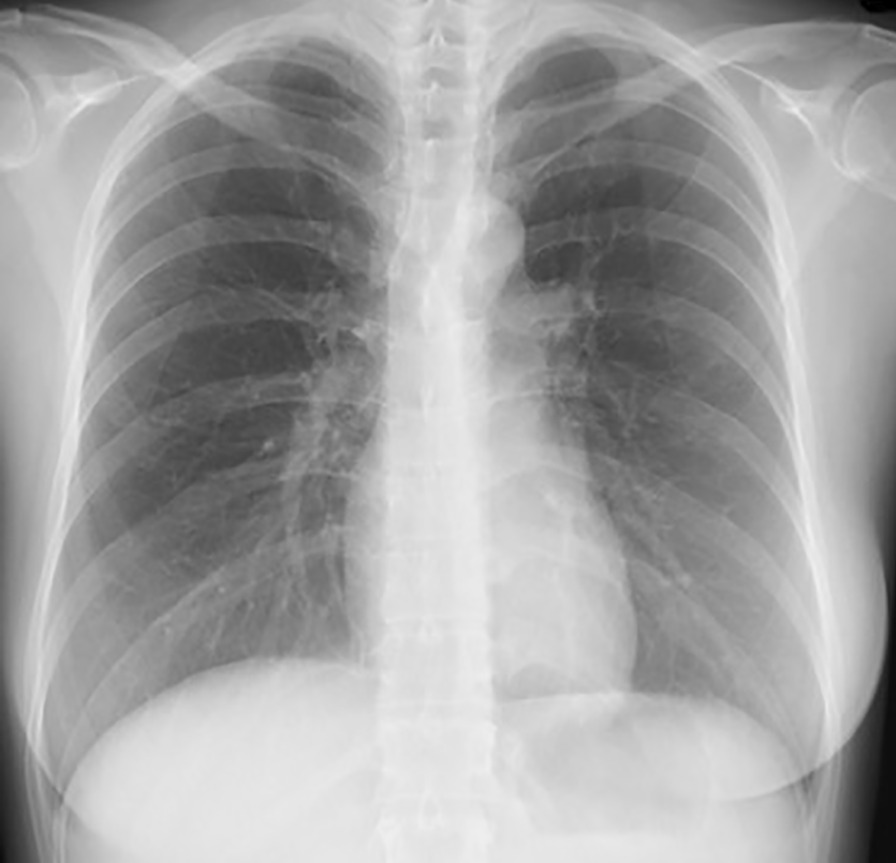
The chest X-ray taken in a standing position 5 days after leaving the intensive care unit. The enlarged pulmonary vascular shadows were improved

A 36-year-old Japanese pregnant woman (gravida 3, para 1) at 28 weeks and 6 days of gestation was admitted to a hospital for threatened preterm labor. Her past medical history was unremarkable. The patient received tocolytic therapy with intravenous ritodrine hydrochloride. Her uterine contractions increased and she was referred to our hospital at 29 weeks and 1 day of gestation. We administered intravenous magnesium sulfate and oral nifedipine as tocolytic therapy and betamethasone for fetal maturation. Twelve hours after arrival at our hospital, she developed dyspnea and her oxygen saturation (SpO_2_) fell to 88% on room air; therefore, oxygen administration was initiated. A chest X-ray revealed enlarged pulmonary vascular shadows (Fig. 1), diagnosed as pulmonary edema induced by tocolytic agents. The patient’s blood pressure did not increase; therefore, pulmonary edema associated with preeclampsia was negative. On the other hand, the volume of infusion was approximately 1000 ml per day due to the administration of ritodrine hydrochloride and magnesium sulfate, and thus overhydration was a possibility. Consequently, administration of ritodrine hydrochloride and magnesium sulfate was stopped. After 2 days on 80% oxygen via a non-rebreather mask, the patient’s arterial blood gas results revealed oxygen tension (PO_2_) of 57 mmHg, pH of 7.46, and carbon dioxide tension (PCO_2_) of 20 mmHg, and she was diagnosed with type 1 respiratory failure. An echocardiogram showed a normal ejection fraction and a D-dimer test was negative; thus, we excluded cardiac insufficiency and pulmonary embolism. Fetal heart rate monitoring was reassuring, and the fetal biophysical profile score was 8 out of 10 points, indicating normal amniotic fluid volume. Therefore, we decided to continue her pregnancy. She was transferred to the intensive care unit (ICU) for advanced respiratory management. NPPV and furosemide were initiated to treat pulmonary edema. During the next 3 days, her respiratory status dramatically improved; before the introduction of NPPV, the SpO_2_ level was 93% or less even with oxygen, but after the introduction of NPPV, the SpO_2_ level was maintained at 97% or more. In addition, her dyspnea was alleviated, and then, even with a reduced oxygen supply, the SpO_2_ level maintained at 97% or more, and the pulmonary vascular shadows observed by chest X-ray also shrank. Eventually, she was weaned off of NPPV, requiring only nasal cannulation. We monitored her fetal heart rate every day for reassurance while she was in the ICU. She left the ICU at 29 weeks and 6 days of gestation, but the next day her uterine contractions were difficult to control. Finally, she underwent a cesarean section due to breech presentation at 30 weeks and 0 days of gestation. She delivered a healthy, 1292 g female infant whose APGAR score was 7 at 1 minute and 8 at 5 minutes. The patient’s chest X-ray was improved (Fig. 2), and she was discharged on day 7 after delivery. The infant needed continuous positive airway pressure for 4 days. The infant’s development was satisfactory and she was discharged on day 55.

## Discussion

To the best of our knowledge, this case is the first report of acute pulmonary edema due to tocolytic agents during pregnancy that was successfully managed with NPPV. In addition, by initiating NPPV before parturition, we succeeded in extending gestation by 5 days. This increase in gestational duration may contribute to better neonatal health outcomes in cases of threatened preterm labor [3, 4].

There are numerous causes of acute pulmonary edema: cardiogenic hydrostatic edema, preeclampsia, acute hemorrhage, sepsis syndrome, pneumonitis, vigorous intravenous fluid therapy, pancreatitis, and tocolytic agents [2]. In addition, diabetes mellitus is a synergistic risk factor with hypertension that can lead to acute heart failure, which may manifest as acute pulmonary edema during pregnancy [5]. Although less commonly utilized worldwide today, tocolytic therapy with β-mimetic drugs was at one time the cause of up to 40% of pulmonary edema [6, 7]. Long-term tocolysis with ritodrine hydrochloride has been implemented in patients and is associated with pulmonary edema [8]. Combination therapy with magnesium sulfate or simultaneous administration of corticosteroids to induce fetal maturation are even more causative of pulmonary edema [2]. A report of pulmonary edema caused by calcium blockers such as nicardipine has also been published [9]. In our case, ritodrine hydrochloride and magnesium sulfate administration increased fluid volume, which may have caused overhydration. Physicians should consider the risk of acute pulmonary edema during treatment for threatened preterm labor.

Several case reports and small series have shown favorable outcomes of NPPV for hypoxemic respiratory failure in pregnancy. In some cases, NPPV during pregnancy improved hypoxemic respiratory failure before delivery, and the baby was delivered at term [10–12]. However, the etiologies were not pulmonary edema induced by tocolytic agents, but severe pneumonia [10], acute respiratory distress syndrome (ARDS) induced by transfusion-related acute lung injury [11], and ARDS induced by all-trans retinoic acid syndrome [12]. Another case series demonstrated that NPPV was effective in delaying delivery for patients with preeclampsia-induced pulmonary edema [13].

Two case reports describe pulmonary edema induced by tocolytic agents, and in those cases, NPPV was initiated during vaginal delivery [14] or after cesarean section [15]. In both cases, NPPV was initiated during or after delivery, such that prolongation of gestational duration was not a goal.

In the present case of acute pulmonary edema induced by tocolytic agents, the risk of pulmonary edema was high because ritodrine hydrochloride, magnesium sulfate, and betamethasone were used. NPPV was initiated because respiratory failure worsened even after ritodrine hydrochloride and magnesium sulfate administration was stopped. NPPV improved respiratory status before delivery. Although the patient delivered prematurely because uterine contractions were difficult to control after she left the ICU, we were able to increase gestational duration by 5 days.

After the initiation of NPPV, physicians should pay attention to the timing of intubation or termination. Pregnancy should be terminated when respiratory failure worsens despite NPPV management or fetal well-being deteriorates.

In conclusion, NPPV can be safely administered to pregnant women with hypoxemic respiratory failure due to tocolytic agent-induced pulmonary edema. NPPV may result in increased gestational duration by stabilizing the respiratory condition, thereby delaying delivery.

## Data Availability

The data that support the findings of this study are available from the medical records. Data are also available from the authors upon reasonable request.

## Abbreviations

NPPV: Noninvasive positive-pressure ventilation
NIV: Noninvasive ventilation
SpO_2_: Oxygen saturation
ICU: Intensive care unit
ARDS: Acute respiratory distress syndrome

## References

[1] Dennis AT, Solnordal CB. Acute pulmonary oedema in pregnant women. Anaesthesia. 2012;67:646–59. 10.1111/j.1365-2044.2012.07055.x 22420683

[2] Cunningham FG, Leveno KJ, Bloom SL, Dashe JS, Hoffman BL, Casey BM, et al editors. Williams obstetrics. 25th ed. New York: McGraw-Hill Education; 2018.

[3] Fanaroff AA, Stoll BJ, Wright LL, Carlo WA, Ehrenkranz RA, Stark AR, et al. Trends in neonatal morbidity and mortality for very low birthweight infants. Am J Obstet Gynecol. 2007;196:147.e1-147.e8. 10.1016/j.ajog.2006.09.014. 17306659

[4] Stoll BJ, Hansen NI, Bell EF, Shankaran S, Laptook AR, Walsh MC, et al. Neonatal outcomes of extremely preterm infants from the NICHD neonatal research network. Pediatrics. 2010;126:443–56. 10.1542/peds.2009-2959 PMC2982806 20732945

[5] Lee KW, Ching SM, Hoo FK, Ramachandran V, Chong SC, Tusimin M, et al. Factors associated with poor-to-moderate quality of life among pregnant women with gestational diabetes mellitus: a cross-sectional study in Malaysia. Qual Life Res. 2020;29:2725–36. 10.1007/s11136-020-02532-3. 32430781

[6] DiFederico EM, Burlingame JM, Kilpatrick SJ, Harrison M, Matthay MA. Pulmonary edema in obstetric patients is rapidly resolved except in the presence of infection or of nitroglycerin tocolysis after open fetal surgery. Am J Obstet Gynecol. 1998;179:925–33. 10.1016/s0002-9378(98)70190-5 9790372

[7] Jenkins TM, Troiano NH, Graves CR, Baird SM, Boehm FH. Mechanical ventilation in an obstetric population: characteristics and delivery rates. Am J Obstet Gynecol. 2003;188:549–52. 10.1067/mob.2003.68 12592270

[8] Shigemi D, Aso S, Yasunaga H. Inappropriate use of ritodrine hydrochloride for threatened preterm birth in Japan: a retrospective cohort study using a national inpatient database. BMC Preg Childbirth. 2019. 10.1186/s12884-019-2352-1. PMC6582481 31215479

[9] Serena C, Begot E, Cros J, Hodler C, Fedou AL, Nathan-Denizot N, et al. Nicardipine-induced acute pulmonary edema: a rare but severe complication of tocolysis. Case Rep Crit Care. 2014. 10.1155/2014/242703. PMC4156995 25215245

[10] Mazlan MZ, Ali S, Abidin HZ, Mokhtar AM, Mukmin LA, Ayub ZN. Non-invasive ventilation in a pregnancy with severe pneumonia. Respir Med Case Rep. 2017;21:161–3. 10.1016/j.rmcr.2017.05.002 PMC5435593 28560149

[11] Gibbs J, Bridges F, Trivedi K, Vullo J. Spontaneous rectus sheath hematoma in pregnancy complicated by the development of transfusion related acute lung injury: a case report and review of the literature. AJP Rep. 2016;6:e325. 10.1055/s-0036-1593353. PMC5025306 27651980

[12] Bassani MA, de Oliveira AB, Oliveira Neto AF. Noninvasive ventilation in a pregnant patient with respiratory failure from all-trans-retinoic-acid (ATRA) syndrome. Respir Care. 2009;54:969–72. 10.4187/002013209793800466 19558746

[13] Hamada K, Chigusa Y, Kondoh E, Ueda Y, Kawahara S, Mogami H, et al. Noninvasive positive-pressure ventilation for preeclampsia-induced pulmonary edema: 3 case reports and a literature review. Case Rep Obstet Gynecol. 2018. 10.1155/2018/7274597. PMC6114237 30186649

[14] Perbet S, Constantin JM, Bolandard F, Vignaud M, Gallot D, Chanseaume S, et al. Non-invasive ventilation for pulmonary edema associated with tocolytic agents during labour for a twin pregnancy. Can J Anesth. 2008;55:769–73. 10.1007/BF03016350 19138917

[15] Fujita N, Tachibana K, Takeuchi M, Kinouchi K. Successful perioperative use of noninvasive positive pressure ventilation in a pregnant woman with acute pulmonary edema. Masui. 2014;63:557–60. 24864580

